# Drivers of cost differences between US breast cancer survivors with or without lymphedema

**DOI:** 10.1007/s11764-019-00799-1

**Published:** 2019-08-24

**Authors:** Lorraine T. Dean, Yusuf Ransome, Livia Frasso-Jaramillo, Shadiya L. Moss, Yuehan Zhang, Kimlin Ashing, Gerald V. Denis, Kevin D. Frick, Kala Visvanathan, Kathryn H. Schmitz

**Affiliations:** 1 Department of Epidemiology, Johns Hopkins Bloomberg School of Public Health, Johns Hopkins University, 615 N Wolfe St, E6650, Baltimore, MD 21205 USA; 2 Department of Oncology, Johns Hopkins School of Medicine, Baltimore, MD USA; 3 Department of Social & Behavioral Sciences, Yale School of Public Health, New Haven, CT USA; 4 Department of Health Policy & Management, Johns Hopkins Bloomberg School of Public Health, Johns Hopkins University, Baltimore, MD USA; 5 Department of Epidemiology, Mailman School of Public Health, Columbia University, New York, NY USA; 6 Department of General Internal Medicine, University of Pennsylvania Perelman School of Medicine, University of Pennsylvania, Philadelphia, PA USA; 7 City of Hope Comprehensive Cancer Center, Division of Health Equities, City of Hope, Duarte, CA USA; 8 Departments of Medicine and Pharmacology, Medicine and Pharmacology, BU-BMC Cancer Center, Boston University School of Medicine, Boston, MA USA; 9 Johns Hopkins Carey Business School, Johns Hopkins University, Baltimore, MD USA; 10 Department of Public Health Sciences, Pennsylvania State University College of Medicine, Pennsylvania State University, Hershey, PA USA

**Keywords:** Breast cancer, Lymphedema, Financial toxicity, Economic burden

## Abstract

**Purpose:**

Breast cancer–related lymphedema is an adverse effect of breast cancer surgery affecting nearly 30% of US breast cancer survivors (BCS). Our previous analysis showed that, even 12 years after cancer treatment, out-of-pocket healthcare costs for BCS with lymphedema remained higher than for BCS without lymphedema; however, only half of the cost difference was lymphedema-related. This follow-up analysis examines what, above and beyond lymphedema, contributes to cost differences.

**Methods:**

This mixed methods study included 129 BCS who completed 12 monthly cost diaries in 2015. Using Cohen’s *d* and multivariable analysis, we compared self-reported costs across 13 cost categories by lymphedema status. We elicited quotes about specific cost categories from in-person interviews with 40 survey participants.

**Results:**

Compared with BCS without lymphedema, BCS with lymphedema faced 122% higher mean overall monthly direct costs ($355 vs $160); had significantly higher co-pay, medication, and other out-of-pocket costs, lower lotion costs; and reported inadequate insurance coverage and higher costs that persisted over time. Lotion and medication expenditure differences were driven by BCS’ socioeconomic differences in ability to pay.

**Conclusions:**

Elevated patient costs for BCS with lymphedema are for more than lymphedema itself, suggesting that financial coverage for lymphedema treatment alone may not eliminate cost disparities.

**Implications for Cancer Survivors:**

The economic challenges examined in this paper have long been a concern of BCS and advocates, with only recent attention by policy makers, researchers, and providers. BCS identified potential policy and programmatic solutions, including expanding insurance coverage and financial assistance for BCS across socioeconomic levels.

## Introduction

The economic burden of cancer treatment and its consequences, termed financial toxicity [1], remains high for breast cancer survivors (BCS) in the United States (US) [2–9]. Direct out-of-pocket costs after active treatment can range from $393 to $561 per month for working-aged BCS in the initial 3 years after treatment [10], and indirect costs amount to $1407 to $2293 per year [11]. Among low-income women, costs can be up to 98% of one’s annual earnings, even with health insurance [3]. Healthcare costs are even higher for survivors who have long-term adverse effects of cancer treatments [12–18], such as breast cancer–related lymphedema [19–22]. Breast cancer–related lymphedema (BCRL) is a chronic condition that affects up to 35% [23, 24] of the 3.5 million BCS in the US (2016) [25]. BCRL is an inflammatory condition that arises when there is disruption of lymphatic flow. Breast cancer surgery, adjuvant treatment, infection, or other trauma to the lymphatic system [24, 26] can lead to a buildup of lymphatic fluid, usually in the arms, breast, and torso [27]. The gold standard of care for lymphedema includes compression therapy (compression garments, banding) and physical therapy [28]. Greater BCRL severity is associated with low socioeconomic position [29], making affordability of BCRL care a critical concern. As a chronic condition, it may have sustained effects on finances long after treatment [30] and may even influence consumer credit [21]. Yet, costs for BCRL are largely understudied in the US context [19, 30].

Our previous analysis of costs for BCS in the US an average of 12 years after cancer surgery suggested significantly higher annual out-of-pocket health and wellness costs for BCS with BCRL compared with those without BCRL [30]. That study also found that only about half of the higher cost for BCS with BCRL was due to BCRL-specific needs such as specialty lotions, therapy visits, complementary and alternative therapies, compression garments and bandaging. Thus, there is a gap in understanding what, beyond BCRL-specific needs, contributes to higher costs among BCS with BCRL compared with BCS without BCRL. This follow-up analysis was conducted to detail the specific types of costs that BCS with or without BCRL face, and to understand why, beyond having BCRL, costs differ among these groups.

## Methods

### Sample

The current study is a follow-up study, for which details of study recruitment have been previously published [21]. From May to September of 2015, 258 women were screened by phone for eligibility in the Physical Activity and Lymphedema Social Economic and Quality of Life (PAL SEQL) follow-up study. Recruits were identified from two sources: prior participants of the (PAL) trial (*n* = 295) [31, 32] who were still alive; or participants who were ineligible (*n* = 163) for the then ongoing Women in Steady Exercise Research (WISER) Survivor Study [33–35], but met requirements for entry into PAL. All participants who had agreed to be contacted about future studies and had up-to-date contact information were contacted. Eligibility criteria included the following: women with Stages I–III invasive breast cancer after completion of active treatment; > 1 lymph node removed, and current residents of Pennsylvania or New Jersey. Patients with active cancer or who were pregnant or planning to become pregnant in the next 6 months were excluded. Of potential participants screened, 37 were ineligible, and 96 declined or dropped out due to lack of time to commit to a longitudinal study, leaving 129 women who were enrolled in the study. Of these, 40 were selected for a qualitative interview using purposeful sampling [36] to ensure representation across BCRL status, age group (over 65 and under 65), and socioeconomic position (using education level as a proxy). After listing participant IDs for each demographic group, we randomly selected at least 10 from each group to invite to an interview; thus, a single interviewee could represent multiple demographics (e.g., over 65 and low socioeconomic position).

### Ethical considerations and informed consent

The Institutional Review Board of the University of Pennsylvania approved the parent study. Written informed consent was obtained from all individual participants.

## Measures

### Demographics

Participants self-reported current age and US census-defined race. Socioeconomic position (SEP) measures included self-reported education, total annual household income before taxes in 2014, total summed value of financial assets, and retirement status. Because financial status is a sensitive topic and subject to high non-reporting [37], income and cash assets were collected as category measures. Self-reported health insurance was classified as public (Medicaid or Medicare), private, or none with some participants reporting both public and private insurance, which were counted in both categories.

### Direct out-of-pocket costs and indirect costs over 12 months (cost diary)

The data collection instrument developed was based on the Goossens’ cost diary, a validated tool for collecting out-of-pocket cost data [38]. Details on this method are documented elsewhere [20] and summarized in the “data collection” section. Direct costs included co-payments for outpatient physician office visits, physical and occupational therapy visits, complementary and integrative therapy visits, emergency department visits, hospitalizations, labs, x-rays and tests; wellness resources (e.g., gym memberships); BCRL-specific healthcare needs (compression garments, bandages); medications or other health-related product that a participant identified; and health insurance premiums if paid out-of-pocket. Other out-of-pocket costs included payments for pain relief medicine, transportation, and nutritional and vitamin supplements. We used an opportunity cost approach [39] to assess indirect costs for lost work or activity hours, and lost assets due to payments for caregiving. Indirect costs were assessed with two questions on (a) number of days which they were unable to perform usual activities; and (b) number of hours of help they needed to carry out daily activities. Participants with BCRL received a version of each question that specified whether the additional help was needed “due to lymphedema”. Patients who reported needing help around the house also reported the number of hours they had paid for domestic assistance. To calculate a monetary equivalent for opportunity costs of lost work, the adjusted mean number of missed workdays was multiplied by the median hourly wage ($16.87) for a 6-h workday based on the US Bureau of Labor Statistics [40] estimates and recommendations for calculating work losses. To calculate the monetary equivalent for domestic assistance, the mean number of hours was multiplied by the daily household productivity rate ($43.37) [41] adjusted to the Consumer Price Index [42].

### Cancer history and treatments

Participants self-reported completion of chemotherapy and/or radiation therapy and/or hormone therapy after cancer surgery, year of breast cancer diagnosis, and cancer stage at diagnosis. Self-report of breast cancer treatment has been validated as over 90% congruent with medical records [43]. We modeled the total number of types of treatments, which is more important to cost than the details of treatment.

### Health conditions and lymphedema

Participants self-reported any of 23 comorbidities and previous diagnosis of breast cancer–related lymphedema (BCRL). To measure upper body BCRL severity, inter-limb volume difference measurements between the affected and unaffected arms were taken using perometry (Juzo, Germany) and were adjusted for humidity, barometric pressure, and time of day. Women who wore compression garments were asked to remove them for at least 1 h prior to perometry assessment.

### Data collection

The same set of data collection procedures were used in this follow-up study as in the parent study [21, 30]. At baseline, participants completed a demographic and health history survey, including report of previous BCRL diagnosis by a health professional, followed by monthly cost diaries. At the start of the study period, participants reported direct and indirect (opportunity) costs related to their overall healthcare [44] for the past 3 months. Then, for each of the next 6 months, participants prospectively reported costs. For the final 3 months, participants estimated projected costs to capture any annual or scheduled visits. This procedure yielded a total of 12 months of cost data. Participants who did not complete a cost diary in a particular month were not included in that month’s analysis and offered no contributing data to that month’s cost estimates. At the end of the 12-month period (November 2015 to January 2016), a subset of survey respondents participated in qualitative interviews, constituting an explanatory sequential design [45, 46]. This approach entails first collecting quantitative data and then collecting qualitative data to inform and provide context for quantitative findings. The standardized semi-structured interview guide (see Appendix of [30]), developed by the study principal investigator (PI), included questions on economic challenges, supports utilized, patient perception of the most challenging economic event since their cancer diagnosis, lasting impact of economic burden, and resource gaps after participants’ breast cancer diagnosis. The study PI (LTD) and a trained research assistant (SLM) conducted interviews. Interviews lasted approximately 15 to 30 min and were conducted in private rooms. Transcripts were de-identified and transcribed verbatim. Quantitative survey data and qualitative interview data were linked through participant study IDs.

#### Data analysis

##### Quantitative component

Demographic characteristics were compared for participants with BCRL versus without BCRL using Chi-squared test with Fischer’s statistic for demographic categories with less than 5 respondents, and non-parametric rank sum tests for non-normally continuously distributed variables. In contrast with the parent study [30], this follow-up analysis focused on how and why costs differed by BCRL status for specific cost categories. Thus, the current study reports unadjusted cost estimates and differences due to smaller sample sizes within the cost categories. Monthly costs by category were compared using Cohen’s *d* and bootstrapped estimates with 200 replications with a seed of (111), allowing us to obtain confidence intervals for non-normally distributed data.

Since our previous analysis estimated that only half of cost differences were due to BCRL costs, the next step of the current analysis sought to understand what drove cost differences between BCS with and without BCRL. We explored this in two steps, focusing only on cost categories that were significantly different by BCRL status. For each category, we generated two models using generalized estimating equations (GEE) with negative binomial distributions. Model 1 used BCRL as an exposure and adjusted for income because it was the only sociodemographic variable that was significantly different by BCRL status. If Model 1 showed BCRL to be significantly related to costs for that category, a second model was generated further adjusting for socioeconomic clinical characteristics. Model 2 gave insight on which variables, above and beyond BCRL status, could potentially account for cost differences. Our previous work suggested that, after adjusting for years since cancer diagnosis, race and age would not be significant predictors in this sample [29] and thus were not included in the multivariable models.

##### Qualitative analysis

Verbatim interview transcripts were input into MAXQDA software program for qualitative analysis. The same qualitative methods and codebook were used for this follow-up study and its previous parent study [30]. First, structural codes were identified based on the domains of economic burden after cancer [44]; then, we added codes based on what emerged from the interviews. The research team organized these codes into a codebook, with subcodes for explanations of specific cost categories. The same text segment could have multiple codes. Each fifth transcript was coded by two analysts. Discrepancies were discussed and resolved among the research team. We then divided the transcripts into groups by BCRL status and compared quotes related to each cost category for each group. Representative quotes are reported to illustrate key findings.

## Results

The 129 participants who contributed data to the quantitative analysis are described in Table 1. The mean age was 63 and the average time since cancer diagnosis was 12 years. Just under half (46.5%) of the participants were diagnosed with BCRL. There was no statistically significant difference by BCRL status in mean age, race, education, wealth, retirement status, and type of insurance. A significantly greater percentage of BCRL were in a lower income category (*p* = 0.02) compared with those without BCRL. Cancer stage at diagnosis, type of adjuvant treatments, and number of comorbidities did not differ by BCRL status. Those with BCRL were on average 3 years farther out from diagnosis (*p* = 0.002) and had greater inter-limb difference (*p* < 0.001). Of the 40 participants who completed qualitative interviews, 24 (60%) had BCRL; the interviewee subset had a greater proportion of high school–educated participants than the overarching quantitative study participant pool.

**Table 1:** Participant baseline characteristics

*N* = 129	BCRL* Yes *n* = 60 (46.51%)	BCRL* No *n* = 69 (54.49%)	*p* value	Interviewees *N* = 40
Demographics				
Age in years, M (SD)	65 (8)	62 (8)	0.11	64 (8)
Race			0.32	
White	35 (57.4)	41 (60.3)		21 (52.5)
Black	24 (39.3)	26 (38.2)		17 (42.5)
Other	2 (3.3)	0 (0.0)		2 (5)
Education completed			0.35	
High school	17 (27.9)	13 (19.1)		19 (47.5)
College	26 (42.6)	29 (42.7)		12 (30)
Graduate school	17 (27.9)	26 (38.2)		9 (22.5)
Income			0.02	
≤ $30,000	8 (13.1)	11 (16.2)		4 (10.5)
$30,001–$70,000	30 (49.2)	18 (26.5)		22 (57.8)
> $70,000	19 (31.2)	35 (51.5)		12 (31.6)
Total cash assets			0.60	
≤ $4,999	17 (27.9)	16 (23.5)		12 (35.1)
$5,000–$49,999	16 (26.2)	13 (19.1)		10 (27.0)
$50,000–$499,999	13 (21.3)	19 (27.9)		10 (27.0)
≥ 500,000	9 (14.8)	13 (19.1)		4 (10.8)
Retired	22 (36.1)	15 (22.1)		10 (25.0)
Insurance type			0.08	
Public	21 (34.4)	19 (27.9)	0.43	12 (30)
Private	49 (80.3)	53 (77.9)	0.74	33 (82.5)
None	1 (1.6)	2 (2.9)	0.62	0
Cancer diagnosis and treatment variables				
Cancer stage at diagnosis			0.09	
Stage 0	9 (14.8)	10 (14.7)		10 (32.3)
Stage 1	11 (18.0)	22 (32.4)		9 (29.0)
Stage 2	11 (31.2)	19 (16.2)		7 (22.6)
Stage 3	9 (14.8)	6 (8.8)		5 (16.1)
Missing	13 (21.3)	19 (27.9)		9 (22.5)
Years since cancer diagnosis (SD)	13 (6)	10 (3)	0.002	12 (5)
Number of adjuvant treatment modalities (SD)	2 (1)	2 (1)	0.13	
Radiation	51 (83.6)	53 (77.9)	0.42	33 (82.5)
Chemotherapy	51 (83.6)	46 (67.7)	0.05	30 (76.9)
Hormonal therapy	29 (47.5)	34 (50)	0.79	10 (25)
Comorbidities	1 (1)	1 (1)	0.46	2 (1)
Inter-limb difference (%)	9.3 (13.4)	− 0.8 (6.1)	< 0.001	7.7 (15.0)

*BCRL breast cancer–related lymphedema

As shown in Table 2, participants with BCRL faced 122% higher average monthly direct costs (*d* = − 0.16, 95% CI [− 0.25, − 0.07]). Despite an equal number of average monthly medical visits, costs were statistically higher for women with BCRL for the categories of co-pays (131%; *d =* − 0.15, 95% CI [− 0.24, − 0.05]), medications (46%; *d =* − 0.17, 95% CI [− 0.29, − 0.04]), and other out-of-pocket costs (167%; *d =* − 0.09, 95% CI [− 0.18, − 0.01]); but lower for lotions (− 28%; *d =* 0.29, 95% CI [0.01, 0.57]). No other variables were statistically different by BCRL status based on effect size difference calculations.

**Table 2:** Monthly cost comparisons for breast cancer survivors with lymphedema or without lymphedema

	Average expenses			
Cost category	BCRL* Yes	BCRL* No	% difference	Cohen’s *d*, 95% confidence interval
Number of health provider visits	1.54 (1.9)	1.50 (2.69)	4%	− 0.01, (− 0.14, 0.11)
Total direct costs	$355.0 ($1676.8)	$159.6 ($373.6)	122%	− 0.16, (− 0.25, − 0.07)
Office visit co-pays	$94.2 ($481.8)	$41.0 ($167.3)	131%	− 0.15, (− 0.24, − 0.05)
Alternative treatments	$16.5 ($84.7)	$10.7 ($45.7)	54%	− 0.08, (− 0.21, 0.04)
Labs and X-rays	$12.3 ($92.2)	$7.3 ($72.4)	68%	− 0.06, (− 0.18, 0.06)
Compression garments/bandaging	$350 ($459.3)	N/A	N/A	N/A
Lotions	$20.7 ($23.1)	$28.8 ($30.0)	− 28%	0.29, (0.01, 0.57)
Gym memberships	$38.9 ($35.9)	$35.8 ($30.0)	9%	− 0.08, (− 0.32, 0.15)
Medications	$60.1 ($149.5)	$41.1 ($56.8)	46%	− 0.17, (− 0.29, − 0.04)
Emergency room (in-patient) costs	$18.7 ($37.2)	$15.0 ($47.4)	25%	− 0.14, (− 1.41, 1.12)
Out-patient hospital costs	$14.6 ($38.5)	$4.0 ($12.6)	290%	− 0.48, (− 1.72, 0.76)
Other out-of-pocket costs	$157.6 ($1456.6)	$59.1 ($288.0)	167%	− 0.09, (− 0.18, − 0.01)
Cost for hired help	$106.5 ($115.7)	$103.3 ($104.0)	3%	− 0.03, (− 0.51, 0.45)
Number of hours for hired help	2.77 (13.3)	3.10 (12.69)	− 13%	0.06, (− 0.36, 0.48)
Opportunity costs, work	$124.9 ($666.5)	$191.7 ($747.3)	− 35%	0.10, (− 0.05, 0.25)
Opportunity costs, home	$9.6 ($45.0)	$7.1 ($36.3)	35%	− 0.06, (− 0.22, 0.11)

Cohen’s *d* computed for effect size in the difference in means using bootstrapped estimates with 200 replications and seed (111) to obtain 95% confidence interval. Effect sizes are significant if the confidence interval does not contain zero.

*N/A* no observations in other category for a comparison

*BCRL breast cancer–related lymphedema

Table 3 includes the results for the cost categories from Table 2 that were significantly different by BCRL status. For co-pays, above and beyond BCRL, patients at the middle-income tier experienced greater costs (PR = 1.02, 95% CI [1.01, 1.04]) than those in the lowest income levels. For lotions and medications, BCRL did not explain cost differences, but was largely driven by those with highest incomes spending more on lotions (PR = 2.05, 95% CI [1.20, 3.50]) and medications (PR = 1.01, 95% CI [1.00, 1.02]). For other out-of-pocket costs, having BCRL primarily explained cost differences (PR = 1.01, 95% CI [1.00, 1.02]).

**Table 3:** Prevalence ratios and 95% confidence intervals for drivers of cost differences, above and beyond lymphedema

	Co-pays		Lotions	Medications	Other out-of-pocket costs	
	Model 1 *N* = 905	Model 2 *N* = 722	Model 1 *N* = 168	Model 1 *N* = 456	Model 1 *N* = 905	Model 2 *N* = 722
Lymphedema	*1.01* *(1.00, 1.02)*	1.00 (0.99, 1.01)	0.77 (0.48, 1.21)	1.01 (0.99, 1.02)	*1.01* *(1.00, 1.02)*	1.00 (0.99, 1.01)
Income (ref = ≤ $30,000)						
$30,001–$70,000	*1.01* *(1.00, 1.04)*	*1.02* *(1.01, 1.04)*	1.67 (0.97, 2.87),	1.00 (0.99, 1.01)	1.00 (0.98, 1.02)	0.99 (0.98, 1.01)
> $70,000	1.01 (0.99, 1.03)	1.00 (1.00, 1.00)	*2.05* *(1.20, 3.50)*	*1.01* *(1.00, 1.02)*	1.01 (0.99, 1.03)	1.00 (0.99, 1.02)
Years since cancer diagnosis		0.99 (0.99, 1.00)				1.00 (1.00, 1.00)
Number of adjuvant treatments (ref = 4)						
1		0.99 (0.97, 1.03)				1.04 (0.96 1.13)
2		1.00 (0.98, 1.03)				1.05 (0.97, 1.14)
3		1.01 (0.99, 1.03)				1.05 (0.97, 1.14)
Number of comorbidities		1.00 (1.00, 1.01)				1.00 (1.00, 1.00)
Cancer stage at diagnosis (ref = Stage 0)						
Stage 1		1.00 (0.99, 1.01)				1.01 (0.98, 1.03)
Stage 2		1.01 (0.99, 1.01)				1.01 (0.99, 1.03)
Stage 3		1.00 (0.98, 1.01)				1.00 (0.97, 1.02)

Based on GEE analysis with independent correlation matrix and robust, adjusted standard error estimates for clustering. Italicized values indicate factors that were significant contributors to cost differences. The *N* for each analysis is the total observations over the eight periods in the multivariable analysis. Some of the significant and non-significant point estimates and confidence intervals appear the same due to rounding up to the nearest two significant digits

### Qualitative findings

Table 4 includes quotes from the qualitative interviews that help further explain why selected costs differed by BCRL status. Not all costs were mentioned in qualitative analysis; thus, quotes are given only for those categories that were brought up by participants during qualitative interviews. Key differences that emerged when comparing those with BCRL with those without BCRL included timing of cancer-related costs, adequacy of insurance coverage, and the role of patients’ socioeconomic position in the use or purchase of health needs.

**Table 4:** Representative quotes explaining differences in cost categories by breast cancer–related lymphedema (BCRL) diagnosis

Cost category*	BCRL Yes	BCRL No	BCRL Yes Name, age, (insurance type)	BCRL No Name, age, (insurance type)	Themes
Office visit co-pays	$94.2 ($481.8)	$41.0 ($167.3)	Doris, 83 (public insurance): Well, no. The only thing, you paid a co-pay to your doctor.... And that, that wasn’t bad then...Because back then it was like $25.00. But now co-pay is $50.00. You know, so…In a matter of 10 to 15 years, things have really went up.	Cheryl, 52 (private insurance): Also, I just found that my co-pays, because the insurance I had, was rather expensive. They are, like, thirty dollars a month every time I went to the doctors, which was kind of hard.	For BCRL+, costs are co-pays that are ongoing due to the need for specialty treatments, while for BCRL−, co-pay costs are concentrated closer to time of breast cancer treatment
Compression garments/bandaging	$349 ($459.3)	N/A	Maria, 73 (public and private insurance): My insurance did not cover the garment, and, you know, the sleeve, and the wrappings…that was, like, $300-some dollars just for the sleeve and the garment that I had to wear. And then I had to buy the bandages, the tape to go with the bandages, you know.	N/A	The need for compression garments, bandages, and sleeves contributes to higher overall costs for those with BCRL
Medications	$60.1 ($149.5)	$41.1 ($56.8)	Anjanee, 60 (private insurance): Well I had to take my 401K money and like pay bills, buy medicine...‘cause when you are always worried about, “All right, I got my medicine this month. Let me not take it as prescribed but kind of not take it every day but every other day so that it’ll last me so when I get more money”.... Maria, 73 (public and private insurance): [The most challenging was] economically the cost of the medication that I received during the time of my diagnosis and treatment, the prescriptions. I did not do any therapy at that time, but when I developed the lymphedema, then I did treatment…I was taking Tamoxifen, I believe, at the time, and I took that for, like, three years, and I had to pay for that.	Carol, 69 (private insurance): I had to stop work. I lost income. So additional medication had to be purchased, so additional copays, additional doctors…my income was shortened, so I had to figure out other ways to make things happen. Diane, 73 (public insurance): …so I have been on continuous medications, and I’m dealing with Medicare telling me what I can and cannot take….I’ve actually stopped a couple [of medications] because I could not afford them, so... And then two years ago, I went into the donut hole.	For both BCRL+ and BCRL−, costs of ongoing medications compromise ability to take full medication regimens
Other out-of-pocket costs	$157.6 ($1456.6)	$59.1 ($288.0)	Maria, 73 (public and private insurance): I have Medicare and … the premium for the secondary insurance is over $200 a month, what am I paying, over $2400 a year.... Emma, 65 (public insurance): …The costs of the organic foods and different supplements, and things like that, that everyone was recommending, that ran into some money. Alexandra, 66 (public insurance): I’ve been coming three times a week [for lymphedema therapy], so there’s a small amount I pay for parking…	Diane (public insurance), 73: My supplemental insurance, to help cover the doctors and stuff, is $227 dollars a month, and then your supplemental to cover your drugs is another 45 a month. And of course, Medicare’s not free. I know everybody acts like it is, but it’s not. That was-- the last time I looked, it was 166 bucks every three months. Eve, 52 (private insurance): Because everywhere, people tell me to call the [American Cancer Society]. Call this person. Call that person. Call here to get wigs. Call here to do that-- and it seems like every door that I tried to walk through was shut in my face…. “Oh, no, you do not qualify.”	Across BCRL+ and BCRL−, supplemental insurance is a common expense BCRL+ may have additional transportation costs for ongoing therapy visits or recommended supplements

#### Theme 1: Ongoing costs persist for BCS, but are different by BCRL status

Women with BCRL reported ongoing costs that continued in the long-term, especially for co-pays and compression garments. For example, respondents with BCRL recounted how co-pay prices have increased over time and referred to comparative past and present experiences, while those without BCRL were more likely to discuss the impact of co-pay prices closer to the time of treatment. Additionally, for women with BCRL, other ongoing out-of-pocket direct costs included transportation to ongoing BCRL therapy or purchase of additional health-related foods and supplements to improve overall health and manage BCRL symptoms. Because BCRL is a chronic condition, these costs would be ongoing and life-long. Women with and without BCRL both suggested that medication costs were an ongoing challenge that compromised their ability to take multi-year oral anti-cancer adjuvant treatments.

#### Theme 2: Inadequacy of insurance

Both women with and without BCRL spoke on the inadequacy or high cost of insurance coverage that precluded getting care for BCRL or other comorbid conditions. Patients with BCRL reported BCRL-specific treatments or supplies that may not have been comprehensively covered by existing insurance plans. For example, insurance did not typically cover compression garments, bandages, or sleeves, resulting in several hundred dollars in out-of-pocket costs. One respondent without BCRL described how switching to public insurance that had a more limited formulary complicated her ability to take medications for comorbid conditions. She further described how costs for medications could change due to an insurance “donut hole”, referring to a point when, after having an initial amount of services covered, publicly insured (i.e.., Medicare Part D) patients pay full price for medications until their out-of-pocket maximum is met and insurance coverage resumes. Cost for supplemental insurance contributed to higher out-of-pocket direct costs for BCS in both groups.

#### Theme 3: Socioeconomic position helps and hinders access to post-cancer healthcare

BCS’ socioeconomic position amplified challenges with accessing health care for post-cancer care and BCRL, especially in the categories of medication and other out-of-pocket costs. One respondent without BCRL described having to stop work due to her cancer diagnosis and the resultant loss of income due to lost work made it challenging to afford medications. BCS with BCRL had to concurrently navigate costs for BCRL therapy on top of adjuvant treatments. As a result, BCS with BCRL had to trade off financial reserves initially intended to secure a future of financial stability, such as retirement funds, or to forgo taking medication as prescribed. For out-of-pocket costs, socioeconomic position emerged again as critical to understanding the patient’s experience. Another quote exemplified that patients at higher income levels still faced challenges with affording care, yet were often ineligible for assistance with their needs.

## Discussion

This study is one of the few to examine the economic burden of breast cancer–related lymphedema, a chronic adverse effect of breast cancer surgery, and to explore specifically what contributes to higher costs for BCRL patients, above and beyond BCRL itself. Monthly average costs for breast cancer survivors (BCS) who have BCRL were 122% higher than BCS who do not have BCRL. Five types of costs vary significantly by BCRL status, and BCRL patients reported that higher costs persisted in the long-term: co-pays, lotions, compression garments, medications, and out-of-pocket costs. Study results reinforce previous findings that patients with BCRL continue to face higher costs, in some part, due to BCRL. This analysis extends this finding to show that divergent costs are exacerbated by underlying pre-morbid socioeconomic and insurance challenges.

Our results point to a need to mitigate healthcare costs, including co-pays, medications, and other health needs that may be paid out-of-pocket by BCS across resource levels, especially for BCS with BCRL. Having BCRL was a contributor to higher costs for co-pays and items listed under other out-of-pocket costs. BCS with BCRL paid over double in co-pay costs despite having the same average number of medical visits compared with non-BCRL BCS. This disparity may suggest that BCS with BCRL have higher cost insurance plans, though this would need to be explicitly explored in further research. Lower expenditures on lotions and higher payments for medications were better explained by underlying differences in socioeconomic position than BCRL status. While high socioeconomic position patterned greater access to lotions and medications, interviewees suggested that high socioeconomic position did not fully protect against economic challenges and that women who do not quality for assistance may still need help. For both BCS with and without BCRL, patients reported a need to make trade-offs between healthcare spending and doing what was best for their health. Patients with limited financial resources had to choose between adherence to medically necessary treatments and managing other health and living needs, often resorting to creative ways to access medications. Taken together, we interpret these results to mean that intervening on BCRL costs alone may not be sufficient to mitigate cost differences or overall cost burden and that patients across the socioeconomic spectrum may need relief from economic challenges. Given the ongoing nature of economic challenges, relief may need to be extended well beyond the initial post-treatment stages.

High medical non-cancer costs of nearly $15,000 for the first 2 years of BCRL treatment for working-aged BCS has been previously documented [19], with most of the costs attributed to outpatient visits unrelated to cancer treatment, probable complications of BCRL, or physical therapy. Medications and BCRL management supplies also contributed to higher costs [19] for BCS with BCRL. The present study’s results emphasize that high costs persist even an average of 12 years later and that costs are overall more than double for participants with BCRL. Patients with BCRL discussed the challenges of managing a chronic condition that has ongoing costs. In particular, patients with BCRL described ongoing co-pay costs that recurred over time while patients without BCRL described co-pay costs as being concentrated around time of treatment. Our findings suggest that a BCS’ path to manage their health over time may be set early on in the survivorship period (i.e., cancer treatment recovery phase) and is influenced by their pre-morbid socioeconomic condition.

While monthly out-of-pocket direct cost estimates found in our study are less than the previously estimated $393 to $561 for working-aged BCS [10], previous estimates are based on a time close to cancer diagnosis and treatment, when patients may still have been using adjuvant treatments. In contrast, our study estimates costs for long-term BCS. This difference suggests that, while still elevated, costs may lessen over time. Nonetheless, our results suggest that even at a monthly cost of $160 for BCS without BCRL and $355 for BCS without BCRL, the cascading of costs [30] accumulates over time and may pose strain.

### Research and policy implications

Previous studies have suggested ways to reduce costs for BCS [44, 47–50], including reducing direct medical costs; increasing the affordability and comprehensiveness of insurance coverage; giving patients prompt information on costs and access to social workers, navigators, and support groups knowledgeable about resources to reduce economic burden; expansion of financial aid, employment protective policies, and home healthcare services. While these recommendations are not specific to BCRL, our results suggest that women with BCRL have higher healthcare costs and needs that may not be directly related to BCRL, and thus, reducing both overall and BCRL-specific healthcare costs can help. Offering assistance for medications, in addition to other healthcare needs like transportation and nutritional supplements, may help socioeconomically vulnerable patients better afford care to manage BCRL and other comorbidities. Given the cascading nature of costs for BCS, early financial intervention for patients who are at risk for chronic decline or managing chronic adverse effects could reduce long-term care costs and morbidity. To determine who early financial interventions should target, future studies should develop risk profiles based on social, economic, and biological risk factors to identify who would most benefit from early financial intervention.

Inadequate insurance coverage for BCRL-related care emerged as a challenge for patients with BCRL and without BCRL. Costs for compression garments and bandages were present only for BCS with BCRL, which contribute to higher overall costs per month since, as the qualitative interviews report, they are not always covered by insurance. High costs will cause patients to use compression garments that no longer apply sufficient pressure to manage BCRL [22, 30]. In the current study and prior, patients recommend insurance providers expand coverage to include BCRL self-management care, including compression garments and bandages [50]. Policymakers can impact coverage as well. A 2016 report found that expanding insurance coverage to include comprehensive BCRL treatment in one state had a less than 0.1% impact on the cost of insurance claims. Coverage included products for self-management, which nearly halved the number of office visits patients needed for BCRL treatment, thus reducing patient office visit costs [51]. The Lymphedema Treatment Act, an amendment to Title XVIII of the Social Security Act (Medicare) to cover certain lymphedema compression treatment items as durable medical equipment, has been introduced to Congress several times [52]. Insurance design plans that encourage equity in costs across individual insurance plans, such as value-based designs, could help mitigate cost disparities across BCS [53].

### Limitations

This study contributes to the body of literature examining why costs for BCS who have BCRL may differ from those who do not have BCRL. Dollar amount estimates were based on the experiences of women in Southeastern Pennsylvania and New Jersey in the year 2015 and may not be generalizable to other regions of the United States; regional variations in insurance coverage may cause cost estimates to vary. This study may underestimate actual costs for the 3-month projected cost estimation. Underreporting was addressed by encouraging participants to supply receipts and medical visit bill summaries in lieu of writing them into the cost diaries themselves; and with monthly text-based, e-mail, and phone messaging reminders to complete the cost diaries, yielding a > 90% response rate in each month of data collected. This analysis did not have a comprehensive list of contributing factors; nevertheless, these findings suggest a starting point of potential contributing factors on which future studies could focus.

## Conclusion

Our results call for fresh consideration of enduring high out-of-pocket costs for breast cancer survivors (BCS) who experience adverse effects after cancer treatment. The study results reinforce the idea that there are significant long-term cost implications for BCS who have BCRL and especially BCS who are socioeconomically challenged. To better reduce cost disparities and provide patient relief from added cost burden, improved and sustained financial coverage (e.g., health insurance and financial assistance) for BCS with BCRL should be considered. Patient-level interventions focused on mitigating or lowering costs for adverse treatment effects should consider both the patient’s socioeconomic context and their other healthcare needs. Given the long-term nature of economic challenges for BCS with BCRL, early financial interventions may be warranted to avert long-term economic burden. Upcoming studies should identify social and economic risk profiles of patients who would most benefit from such interventions.
